# Access to primary health care for acute vascular events in rural low income settings: a mixed methods study

**DOI:** 10.1186/s12913-017-1987-8

**Published:** 2017-01-18

**Authors:** Shyfuddin Ahmed, Muhammad Ashique Haider Chowdhury, Md. Alfazal Khan, Nafisa Lira Huq, Aliya Naheed

**Affiliations:** 1 Initiative for Non-communicable Diseases (NCD), Health Systems & Population Studies Division (HSPSD), icddr,b, 68 Shaheed Tajuddin Ahmed Sharani, Mohakhali, Dhaka 1212 Bangladesh; 2 Nutrition & Clinical Service Division (NCSD), icddr,b, 68 Shaheed Tajuddin Ahmed Sharani, Mohakhali, Dhaka 1212 Bangladesh; 3 Maternal and Child Health Division (MCHD), icddr,b, 68 Shaheed Tajuddin Ahmed Sharani, Mohakhali, Dhaka 1212 Bangladesh

**Keywords:** Access to health care, Acute vascular events, Stroke, Myocardial infarction, Bangladesh

## Abstract

**Background:**

Cardiovascular diseases (CVDs) are the leading cause of global mortality. Among the CVDs, acute vascular events (AVE) mainly ischemic heart diseases and stroke are the largest contributors. To achieve 25% reduction in preventable deaths from CVDs by 2025, health systems need to be equipped with extended service coverage in order to provide person-centered care. The overall goal of this proposed study is to assess access to health care in-terms of service availability, care seeking patterns and barriers to access care after AVE in rural Bangladesh. We will consider myocardial infarction (MI) and stroke as acute vascular events.

**Methods/Design:**

We will conduct a mixed methods study in rural Matlab, Bangladesh. This study will comprise of a) health facility survey, b) structured questionnaire interview and c) qualitative study. We will assess service availabilities by creating an inventory of public and private health facilities. Readiness of the facilities to deliver services for AVE will be assessed through a health facility survey using ‘service availability and readiness assessment’ (SARA) tools of the World Health Organization (WHO). We will interview survivors of AVE and caregivers (present and accompanied the person during the event) of person who died from AVE for exploring patterns of care seeking during an AVE. For exploring barriers to access care for AVE, we will conduct in-depth interview with survivors of AVE and caregivers of the person who died from AVE. We will also conduct key informant interviews with the service providers at primary health care (PHC) facilities and government high level officials at central health administration of Bangladesh.

**Discussion:**

This study will provide a comprehensive picture of access to primary health care services during acute cardiovascular events as stroke & MI in rural context of Bangladesh. It will explore available service facilities in rural area for management, utilization of services and barriers to access care during an acute emergency. This study will help to generate hypothesis, develop programs and policies for better access to care for AVE in similar rural settings considering barriers of access and improving utilization.

**Electronic supplementary material:**

The online version of this article (doi:10.1186/s12913-017-1987-8) contains supplementary material, which is available to authorized users.

## Background

Cardiovascular diseases (CVD) -mainly ischemic heart diseases and cerebrovascular diseases (stroke)-are the major contributors to the global burden of diseases and premature mortality worldwide [1]. About 30% of deaths in low- and middle- income countries are due to CVDs [2]. In South Asia, high rates of CVDs are observed at a younger age than in other countries, causing a greater loss of productive life years with severe economic consequences [3]. Bangladesh, a country with a low-income economy of South Asia, has been experiencing a demographic and epidemiological transition with rapid urbanization and globalization and a gradual increase in life expectancy [4, 5]. The common risk factors for CVD, including behavioral, metabolic and physiological risk factors are highly prevalent in Bangladesh [6, 7]. In 2014, 17% of the total deaths in Bangladesh were due to CVD [8]. The high burden of CVD in Bangladesh is confirmed in a recent study which found ischemic heart disease (IHD) and stroke as top two causes of years of life lost (YLLs) in Bangladesh [5].

Among the CVDs, the main clinical burden consists of acute events, although some of the CVDs can cause stable or slowly progressive clinical syndromes, such as stable angina and intermittent claudication. Acute vascular events (AVE) specifically coronary heart diseases [especially myocardial infarction (MI)] and cerebrovascular accident (Stroke) are the leading cause of premature death and disability [9]. The exact incidence of AVE in Bangladesh is not known. Only a limited number of small-scale epidemiological studies reported the prevalence of acute vascular event. It was reported that about 1.85% of rural population had coronary artery diseases [10] and 0.03% had stroke [11]. Data on mortality from AVEs are also limited. It was found that population-attributable mortality of stroke was 25.2% [12], which was based on the verbal autopsy instrument. But in terms of care seeking, it is unknown where Bangladeshi population seek care during AVE, particularly in the rural areas.

The primary health care (PHC) system of Bangladesh has traditionally been focusing on services related to maternal, child and reproductive health, immunization and communicable diseases. Health system of Bangladesh performed well in-terms of family planning, immunization, oral rehydration therapy, maternal and child health, tuberculosis, vitamin A supplementation. It has also achieved some millennium development goals, such as reduction of maternal mortality [13]. The country is also moving towards universal health coverage (UHC). The rising burden of non-communicable diseases (NCD) along with shortage human resource for health, limited access to healthcare for NCDs is challenging Bangladesh health systems [14], which are further imposing challenges to the three dimensions of UHC. Although a dedicated unit has been established for NCD services within the ministry of health and family welfare but access and availability to essential NCD services remains fragmented [14]. To fulfill the objective of UHC, emergency health service coverage up to PHC level has become imperative, as acute NCD events like AVE, particularly MI and stroke, has become top most killers even in lower middle income countries like Bangladesh. To identify gaps and opportunities for further strengthening the local health services, evidence of available access to care and utilization of services for AVE management, especially for rural population, is vital. Information from such evidence can be used to give directions to policy makers on how to manage two fatal diseases at primary care setting and to reduce overall NCD mortality in Bangladesh and other low income settings.

### Objective

The main objective of this study is to evaluate access to health care in-terms of service availability, care seeking patterns and barrier to access care for acute vascular events in rural Bangladesh. We will consider myocardial infarction (MI) and stroke as acute vascular events. The specific objectives are as follows:

1. To explore service availability and readiness of rural primary health facilities for management of acute vascular events.
2. To identify care seeking patterns during an acute vascular event.
3. To explore barriers to access care for acute vascular event at rural primary care facilities.

## Methods/Design

**Fig. 1:**
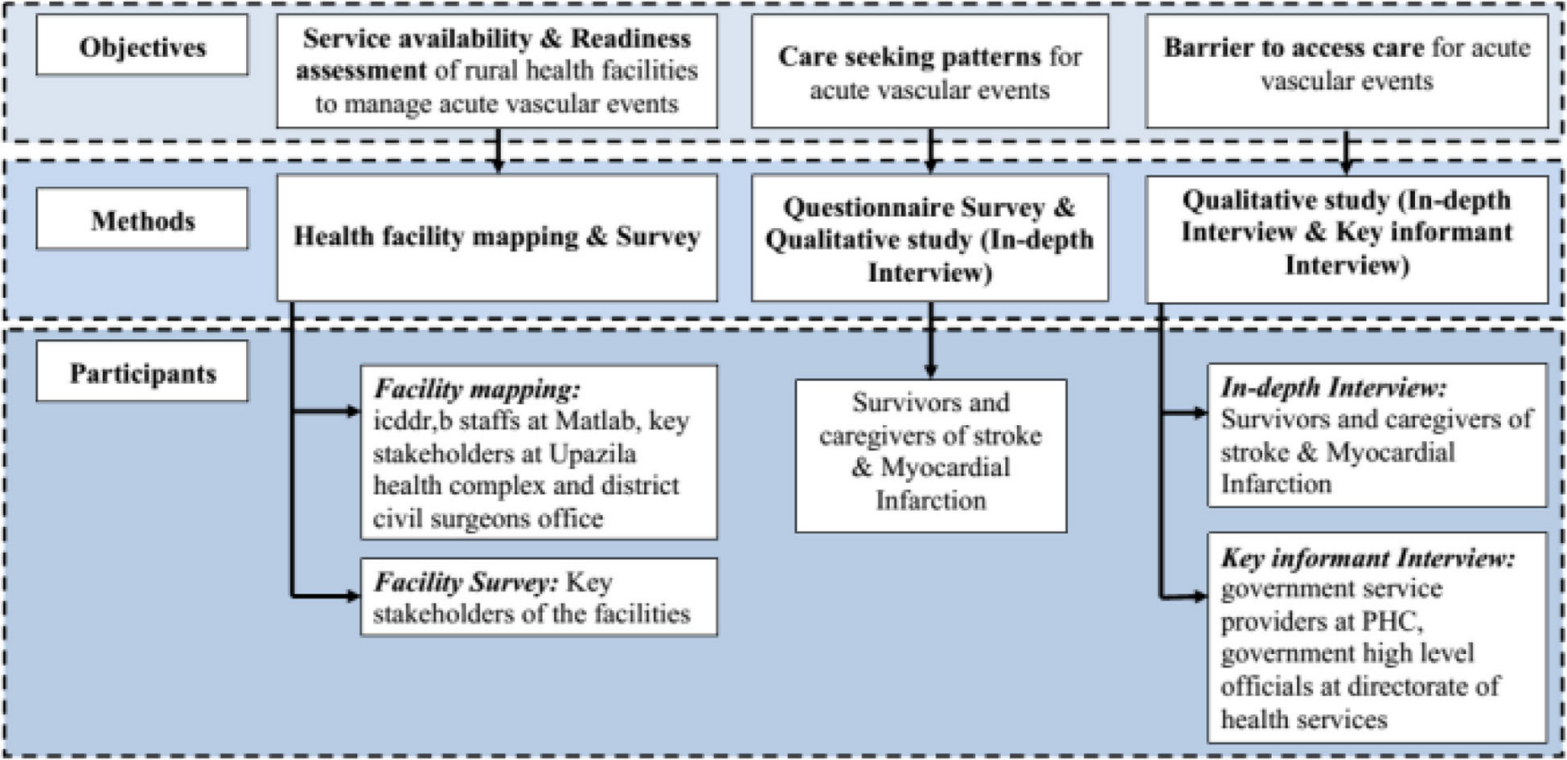
Study Design

This is a mixed methods study, comprising of a health facility survey, survey of care seeking patterns during an AVE, and a qualitative study (Fig. 1).

### Study setting

This study will be conducted in rural Matlab, Chandpur district, Bangladesh. Matlab is located about 55 km southeast of Dhaka, the capital city of Bangladesh. Since 1966, icddr,b has implemented a Health and Demographic Surveillance System (HDSS) in Matlab, which records registration of births, deaths, and migrations, in addition to carrying out periodical censuses [15]. Currently it covers 184 km^2^ and has 142 villages, each with about 1500 people. The total population of Matlab is about 225,000. HDSS is divided into an icddr,b service area and a government service area. People living in both areas have access to usual government primary healthcare services. However, icddr,b service program is available exclusively in icddr,b service area, which includes child and reproductive health services, maternal health services, family planning services and immunization. The community health research workers (CHRWs) of HDSS obtain vital demographic and health information by visiting each household in their assigned areas bi-monthly. A verbal autopsy (VA) questionnaire is administered to any relative who lived with a deceased within 6 to 12 week by trained field research assistants. Verbal autopsies of Matlab HDSS are reviewed by a paramedic to assign cause of death in accordance with the International Statistical Classification of Diseases and Related Health Problems, tenth revision (ICD-10) [15].

### Mapping of health facilities

For assessing service availability, we will map all health care facilities of the study site. The primary motive of this mapping exercise will be to identify health facilities that are accessible to or are serving rural population. We will do it in a two steps process. First, we will develop a comprehensive inventory of all the health facilities of Matlab with the help of relevant stakeholders. We will verify the list and identify other facilities through community mapping. We will invite CHRWs of icddr,b and request them to list and locate any facilities in their working area. Our field staff will visit and confirm the availability of the health facilities and collect Global Positioning System (GPS) readings of the facility. Our second step will be to plot the position of the health facility in the map. To do this, we will use geographic information system (GIS) maps of icddr,b as base maps. This GIS was established in 1994 and is periodically updated with new data. In Matlab surveillance area, GIS collects spatial data through GPS which include locations of baris (cluster of a group of households sharing common yard), tube-wells, ditches, ponds, health facilities, educational institutes, mosques, markets, etc. Our research staff will visit all the health facilities, confirm the location in GIS and collect spatial data through GPS. The final step in the mapping process will be to add a point layer to the base map that consists of GPS coordinates of all the health facilities surveyed by the survey team.

### Health facility survey

We will also conduct a health facility survey consisting of health facility visits, interviewing key stakeholders of the facility and observation of key items. We will use the service availability and readiness assessment (SARA) tool developed by the World Health Organization (WHO) [16]. This tool is designed to assess and monitor the service availability and readiness of the health sector and to generate evidence to support the planning and managing of a health system. This tool helps to generate reliable and regular information on service delivery (such as the availability of key human and infrastructure resources), on the availability of basic equipment, basic amenities, essential medicines, and diagnostic capacities, and on the readiness of health facilities. We will contextualize the SARA tool to assess readiness of the rural health facilities for management of AVE. Proposed context specific tool will be finalized after consultation with a cardiologist. We will identify a key person who is aware of the service and management of all public and private facilities and invite them for an interview. Following proper consenting process from the authority, we will administer SARA questionnaire (Additional file 1). Service readiness of the rural health facilities will consist of both general and service-specific component for management of acute vascular event.

#### Indicators of service readiness

Service readiness reflects the overall capacity of health facilities to provide basic services at minimum standards. Four domains of general service readiness are included in SARA tool and indicators are tracked through tracer item (Table 1). Service-specific readiness for acute vascular events will be illustrated across four domains as described in Table 2. Individual tracer indicator will be summarized as composite measures, namely the proportion of facilities with all tracer items available on the day of the visit.

**Table 1:** Tracer items for general service readiness

Category	Tracer Items
Basic amenities and equipment	Amenities: electric power; improved water source within 500 m of facility; room with auditory and visual privacy for patient consultations; adequate sanitation facilities for clients; communication equipment (phone or short wave radio); computer with email/internet access; emergency transportation. Equipment: weighing scales (child, adult); thermometer; light source; refrigerator.
Standard precautions	Safe final disposal of sharps, safe final disposal of infectious wastes; appropriate storage of sharps, appropriate storage of infectious waste; disinfectant; single-use standard disposable or autodisposable syringes; soap and running water or alcohol-based; hand rub; latex gloves; guidelines
Laboratory testing capacity	Blood haemoglobin; blood glucose; blood smear or rapid test for malaria parasites; a urine dipstick protein; urine dipstick glucose; HIV antibody test; syphilis rapid test; urine pregnancy test
Essential medicines	Availability of Essential drugs supplied by government

**Table 2:** Service specific readiness indicator for service availability and readiness assessment for acute vascular event

Domain	Tracer Items
Staff and guidelines	Specialist/Staff trained management of acute vascular event Guideline on management of acute vascular event
Equipment	Blood pressure apparatus
Diagnostics	ECG Echocardiography
Medicines and commodities	Medicine (aspirin, β-blockers, clopidogril, statin, calcium-channel blockers, long acting nitrates, anticoagulants, alti-ulcerants, dopamine etc.) Non-invasive procedures (Anticoagulant treatment, Thrombolysis etc.)

**Table 3:** Criteria to define Myocardial Infarction and Stroke survivors

Myocardial infarction	Stroke
Confirmed case of MI diagnosed by a physician OR history of following: • Chest pain described as a pressure sensation, fullness, or squeezing in the midportion of the thorax • Radiation of chest pain into the jaw or teeth, shoulder, arm, and/or back • Associated dyspnea or shortness of breath • Associated epigastric discomfort with or without nausea and vomiting • Associated diaphoresis or sweating • Syncope or near syncope without other cause • Impairment of cognitive function without other cause	Confirmed case of Stroke diagnosed by a physician OR history of following: • Have had paralysis or weakness on one entire side of the body, an arm or a leg. • Have out of the corner of his mouth pie, unable to bring it to normal voluntarily. • Have impaired speech, or trouble talking to someone for failing to articulate, pronounce words or sentences correctly. • Have numbness or loss of sensation on one side of the body, an arm or a leg, more than a full day duration.

### Survey to identify care seeking pattern

To identify care seeking pattern during an AVE, we will conduct a questionnaire survey. We want to explore what/who are the first medical contact points during an AVE; who took the decision to seek care; how long it took to reach health facilities; what type of health facility and treatment they received etc. We also want to explore whether there is any difference in pattern of care seeking by fatality of AVE. We will explore care seeking patterns during fatal and nonfatal AVE. We will identify survivor of stroke and MI by a two-step process. In first step we will generate a list of survivors of stroke and MI who had an attack within last 12 months with the help of icddr,b’s CHRWs. They will identify survivors of stroke and MI during their routine household visit using the criteria mentioned in Table 3. In next step, we will administer a structured questionnaire (Additional file 2) among survivors of stroke or MI to identify health seeking patterns during an AVE. Fatal stroke (ICD code: I60-I69) and MI cases (ICD code: I20-I22) will be identified from HDSS verbal autopsy (VA) database. We will identify the households of the deceased individuals who died from stroke or MI and conduct a structured interview with his/her caregiver who was present and accompanied the patient during the event about health seeking patterns during that event.

### Qualitative study

We will carry out a qualitative study for exploring health seeking behavior, understanding personal barriers, policy issues and barriers for expansion of service coverage for AVE at PHC level. A multi-method approach to qualitative data will be undertaken. Data will be obtained from three convenience samples: (a) in-depth interviews with survivors who had a stroke or MI within last 12 months; (b) in-depth interviews with caregivers (person who was present and accompanied the patient during the event) of fatal and nonfatal stroke or MI; and (c) key informant interview of service provider at PHC facilities and government high level officials at directorate general of health services. The recruitment of study participants will continue until data redundancy is reached. Qualitative interview will be conducted using an interview guideline (Additional file 3).

The research team will invite survivors and caregivers of stroke or MI to explore the decision making process, barriers to access care and coping strategies from the receivers’ perspectives. Individual’s perceptions of their needs and their attitude towards quality of services at PHC levels, utilization of PHC during emergency, coping strategies in terms of catastrophic healthcare expenditures and support from the family, financial constrains including fee of services, out of pocket expenditure, charges for specific services for utilizing PHC facilities will also be explored qualitatively. Through key informant interview (KII), we will try to explore barriers to expand service coverage for AVE at PHC level. Service provider’s perceptions of infrastructural barriers including equipment, facilities and services will also be explored through qualitative interview.

### Sample size

We plan to map all the health facilities and conduct health facility survey in all the health facilities in study site to assess service availability and readiness. We want to conduct our survery among 200 survivor and caregivers of storke or MI. Although this study does not involve any hypothesis testing or any parameter estimation, total numbers of the survey sample are based on some parameter estimation. Assuming 50% of the rural population will visit government primary care facilities as a form of first contact point to seek care, we will be requiring 194 samples at 7% percentage point with 95% confidence interval. The rational for assuming 50% of the population is, proportion of 0.5 or 50% indicates the maximum variability in a population, and it is often used in determining a more conservative sample size, that is, the sample size may be larger than if the true variability of the population attribute (26). Outcomes of each objective are presented in Table 4.

**Table 4:** Outcomes according to each objective

Objectives	Outcome
Objective 1	
Facility Mapping	Area map indicating health facilities
Facility survey	Proportion of facilities with key tracer items (Tables 1 and 3)
Objective 2	
Survey to identify health care seeking patter	- type of first medical contact point, - key decision maker for care seeking, - time required to reach hospital, - time required for initiation of treatment, - treatment provided by hospital
In-depth interview	- health seeking behaviors following acute vascular events
Objective 3	
Barrier to access care	- perceived needs, quality of services and accessibility at government primary care facilities during acute vascular events - perceived affordability of the services, coping strategies during an acute vascular events - barriers and facilitators to incorporating care for acute vascular events

### Data analysis

Quantitative data will be collected using structured questionnaires and qualitative interview will follow a guideline. Data collection forms will be checked on a daily basis to ensure complete and accurate data entry by field research workers. Cross checking of data collection forms will be performed in the fields by field supervisor for inconsistency and missing data. The data from quantitative survey and in-depth interview will be analyzed separately but triangulation of data from various sources will be performed.

#### Quantitative data analysis

We will use descriptive statistics to summarize data as percentages or mean & SD, or as median when values are not normally distributed. We will compare continuous variables among different relevant categories using Student’s t-tests and discrete variables using Chi-squared tests. We will try to explore correlations between mortality (from Stroke and MI) with different socioeconomic variables, exposure to risk factors, type of first medical contact, proximity of the health facilities from the household, time to reach hospitals, time to initiation of treatment, availability of emergency services in the facilities etc. We will analyze data using SPSS Version 20 and Microsoft Excel.

#### Qualitative data analysis

Qualitative data will be recorded in digital recorder. We will transcribe recorded interviews in verbatim as soon as they are available. Transcriptions will then be translated into English. The research team will then perform content analysis to identify themes that emerged from the in-depth and key informant interviews. The identified themes will be based on perceptions and understanding of the information. Once transcripts are coded, reports will be generated to address the research objectives. The results will be summarized and presented according to the context and some data will be presented verbatim to substantiate or reflect more important views and ideas.

## Discussion

This study will provide a comprehensive picture of access to PHC services during an acute emergency in rural context of Bangladesh. It aims to explore available service facilities in rural area for management of AVE, utilization of services during an emergency and barriers to access care. This study will help to generate hypothesis, develop programs and policies for better access to care for AVE in rural settings considering barriers of access and improving utilization. Facilitating access is concerned with helping people to command appropriate health care resources to preserve or improve their health [17].

The prevention, treatment, and management of chronic diseases require a core range of interventions that include primary preventions, proactive case findings (e.g., assessment of risk factors and screening), education of both the public and health-care workers, efficient referrals, pharmacological and psychosocial interventions, long-term surveillance and, monitoring and assessment of quality of care [18]. To achieve the World Heart Federation’s target of 25% reduction in preventable deaths from CVDs by 2025, health systems need to be transformed to provide person-centered care with improved outreach and self management to effectively manage risk factors, illness episodes, and multimorbidity. Health facilities in low-income and middle-income countries need to be strengthened to develop reliable individual records that enable assessment and management of risks of individuals under their care [19].

The problem of access to health care is particularly acute in Bangladesh. One crucial determinant of health seeking among rural population, particularly women, is the accessibility of medical care. Barriers to care are usually because of location, financial requirements, bureaucratic responses to the patient, ssocial distance between client and provider, and the sex of providers [20]. It is unknown whether there are any inequalities in terms of access to services, care seeking and health outcomes of acute catastrophic events where immediate primary care is needed for avoidance of preventable complications. It is well evident that reducing delays in access to hospital, and improving affordability of urgent care could reduce morbidity and mortality from AVEs and avert potentially preventable complications [21, 22]. Utilization of health services is a complex behavioral phenomenon that is affected by factors such as availability, distance, costs, quality of care, social structure, and health beliefs [23]. Our mixed methods will explore utilization patterns and barrier for utilization of primary health care services in rural context.

This study has a few limitations. A part of the study site has an ongoing health program of icddr,b which might have an effect on care seeking patters. If there is any such difference in care seeking, we will present data separately. Identification of fatal stroke and MI cases might not be possible in absence of HDSS VA systems. Finally there is a possibility of recall bias as we will be gathering retrospective information.

In summary, to address the problem of premature mortality from AVE such as stroke and myocardial infarction, health systems need to be accessible and affordable, especially for rural population where majority of the population in low income setting reside. To do so, identification of existing opportunities and barrier of access will help to generate hypothesis and guide research.

## Additional files

Additional file 1: Facility Survey questionnaire. (PDF 320 kb)
Additional file 2: Questionnaire to identify care seeking pattern during acute vascular event. (PDF 722 kb)
Additional file 3: Qualitative interview guideline. (PDF 238 kb)
Additional file 4: Consent forms. (DOCX 21 kb)

## Abbreviations

AVE: Acute Vascular Events
CHRW: Community health research workers
CVD: Cardiovascular Diseases
GPS: Global Positioning System
HDSS: Health and Demographic Surveillance System
ICD: International statistical classification of diseases
Icddr,b: International center for diarrheal diseases research, Bangladesh
IHD: Ischemic heart disease
MI: Myocardial Infarction
NCD: Non-communicable Diseases
PHC: Primary health care
SARA: Service availability and readiness assessment
UHC: Universal Health Coverage
VA: Verbal autopsy
WHO: World Health Organization
YLLs: Years of life lost
