# Supplementary material for: Access to primary health care for acute vascular events in rural low income settings: a mixed methods study
Source: BMC Health Serv Res. 2017 Jan 18;17:47. doi: 10.1186/s12913-017-1987-8 (PMC5242000; doi:10.1186/s12913-017-1987-8)
Supplement: Additional file 1: — Facility Survey questionnaire. (PDF 320 kb) [file 12913_2017_1987_MOESM1_ESM.pdf]

Service availability and readiness for management of acute vascular event in rural Bangladesh

Facility survey

Sl #	Question	Code	Direction
	**FACILITY IDENTIFICATION (স্বাস্থ্যকেন্দ্র সনাক্তকরন)**		
1.	Facility Number<br>(স্বাস্থ্য কেন্দ্র নাম্বার)	☐☐☐☐	
2.	Location<br>(স্বাস্থ্য কেন্দ্রর অবস্থান)	icddr,b service area (আইসিডিডিআরবি পরিষেবা এলাকা) .... 1<br>government service area (সরকারি পরিষেবা এলাকা) 2	☐
3.	Type of Facility<br>(স্বাস্থ্য কেন্দ্রর ধরন)	Upazila Health Complex (উপজেলা স্বাস্থ্য কমপ্লেক্স)............ 1<br>Private Clinic (প্রাইভেট ক্লিনিক) .... 2<br>Diagnostic/Consultation Center (ডায়াগনস্টিক / পরামর্শ কেন্দ্র) 3<br>Union Sub-center (ইউনিয়ন স্বাস্থ্য উপ-কেন্দ্র) .... 4<br>Union Health & Family welfare center (ইউনিয়ন স্বাস্থ্য ও পরিবার কল্যান কেন্দ্র) .... 5<br>Community Clinic (কমিউনিটি ক্লিনিক)........................... 6<br>Other (Specify) [অন্যান্য (উল্লেখ করুন] 99	☐
4.	Managing Authority<br>(পরিচালন কর্তৃপক্ষ)	Government/Public (সরকারি) .... 1<br>NGO/Not-For-Profir (এনজিও) .... 2<br>Private-For-Profit (বেসরকারি) ....3<br>Other (Specify) [অন্যান্য (উল্লেখ করুন] 99	☐
5.	Type of services (সেবার ধরন)	Outdoor only (শুধুমাত্র বর্হিবিভাগ) .... 1<br>Indoor only (শুধুমাত্র অভ্যন্তরিন) .... 2<br>Both inpatien & outpatient Services (বর্হিবিভাগ ও অভ্যন্তরিন উভয়ই) 3	☐
	**GEOGRAPHIC COORDINATES (ভৌগলিক স্থানাঙ্ক)**		
6.	**Altitude** (উচ্চতা)	☐☐☐☐ Meters (মিটার)	
7.	**Latitude** (অক্ষাংশ)	**N/S (উত্তর/দক্ষিন) ....**<br>**a** ☐☐ **Degrees/DEC (ডিগ্রি)**<br>**b.** ☐☐ **Minute (মিনিট)**<br>**c.** ☐☐☐ **Second (সেকেন্ড)**	
8.	**Longitude** (দ্রাঘিমাংশ)	**E/W (পূর্ব/পশ্চিম) ....**<br>**a** ☐☐ **Degrees/DEC (ডিগ্রি)**<br>**b.** ☐☐ **Minute (মিনিট)**<br>**c.** ☐☐☐ **Second (সেকেন্ড)**	
	**SERVICE AVAILIBILITY (স্বাস্থ্যসেবার প্রাপ্যতা)**		
9.	**Staffing (স্বাস্থ্য সেবাদানকারি):**<br>Please tell me how many staff with each of the following qualifications are currently assigned to, employed by, or seconded to this facility. Please count each staff member only once, on the basis of the highest technical or professional qualification. For doctors, I would also like to know, of the total number, how many are part-time in this facility. (দয়া করে আমাকে জানাবেন, নিম্নউল্লেখিত স্বাস্থ্যসেবাদানকারিদের মধ্যে, কতজন এই প্রতিষ্ঠানে নিযুক্ত/কর্মরত আছে। সর্বোচ্চ কারিগরী বা পেশাদার যোগ্যতার ভিত্তিতে, শুধুমাত্র প্রতিটি স্বাস্থ্যসেবাদানকারিকে একবার গণনা করুন. ডাক্তারদের ক্ষেত্রে, আমি জানতে চাইব কতজন খন্ডকালিন।)		

		A) Assigned/Employed (নিযুক্ত/কর্মরত)	B) Part Time (খন্ডকালিন)	
	9.1. Generalist (non-specialist) medical doctor (সাধারণ (অ-বিশেষজ্ঞ) ডাক্তার)	☐☐☐	☐☐☐	

	9.2. Specialist Medical doctor (বিশেষজ্ঞ ডাক্তার)	☐☐☐	☐☐☐	
	9.3. Cardiologist (কার্ডিওলজিষ্ট/হৃদরোগ বিশেষজ্ঞ)	☐☐☐	☐☐☐	
	9.4. Neurologist (নিওরোলজিষ্ট/স্নায়ুরোগ বিশেষজ্ঞ)	☐☐☐	☐☐☐	
	9.5. Non-Physician clinicians/Paramedical Professions (প্যারামেডিকস্)	☐☐☐	☐☐☐	
	9.6 Nursing Professionals (নার্স)	☐☐☐	☐☐☐	
	9.7. Pharmacists (ফার্মাসিস্ট)	☐☐☐	☐☐☐	
	9.8. Laboratory technicians (medical and pathology) [ল্যাবরেটরি টেকনিশিয়ান (মেডিকেল ও প্যাথলজি)]	☐☐☐	☐☐☐	
	9.9. Community Health workers (কমিউনিটি স্বাস্থ্যকর্মী)	☐☐☐	☐☐☐	
10.	How many ***inpatient beds*** in total does this facility have (Excluding delivery bed)? [এই স্বাস্থ্যসেবাকেন্দ্রটির আভ্যন্তরিন সেবা কত শয্যা বিশিষ্ট (প্রসবশয্যা ছাড়া)?]	# of inpatient beds ☐☐☐<br>শয্যা সংখ্যা		
	10.1. Of the inpatient beds in this facility, how many are dedicated for cardiac/vascular/neurology emergency? [আভ্যন্তরিন সেবার শয্যাগুলোর মধ্যে কতগুলো শয্যা কার্ডিয়াক বা হৃদরোগ/ রক্তনালী/স্নায়রোগের জরুরী সেবার জন্য বরাদ্দ?]	# of inpatient beds (শয্যা সংখ্যা) ☐☐☐<br>Cardiaovascular (হৃদরোগ/ রক্তনালী) ☐☐<br>Neurology (স্নায়রোগ) ☐☐<br>Emergency (জরুরী সেবা) ☐☐<br>Working Emergency bed (কার্যকরি জরুরী সেবার শয্যা) ☐☐		
11.	Does this facility have a functioning ***land line/cellular telephone*** that is available to call outside at all times client services are offered? [সবসময় রোগীর সেবা প্রদানের জন্য এই স্বাস্থ্যসেবাকেন্দ্রটিতে কোন কার্যকরি টেলিফোন/মোবাইল ফোন আছে কি ?]	Yes (হ্যাঁ) ………………………………….. 1<br>No (না) …………………………………… 2		☐
12.	Does this facility have a ***functional ambulance*** or other vehicle for emergency transportation for clients (Stationed at this facility or operates from this facility)? [রোগীদের জরুরি ভিত্তিতে স্থানান্তরের জন্য এই স্বাস্থ্যসেবাকেন্দ্রে কোন কার্যকারি অ্যাম্বুলেন্স বা অন্য কোন যানবাহন আছে কি? (যা এই স্বাস্থ্যসেবাকেন্দ্রে সার্বক্ষণিক থাকে বা এখানে ব্যবহৃত হয়)]	Yes (হ্যাঁ) ………………………………….. 1<br>No (না) …………………………………… 2		☐
	12.1 Does this facility have access to an ambulance or other vehicle for emergency transportation for clients (Stationed at this facility or operates from this facility)? [প্রয়োজনে রোগীদের জরুরি ভিত্তিতে স্থানান্তরের জন্য এই স্বাস্থ্যসেবাকেন্দ্রের কি কোন কার্যকারি অ্যাম্বুলেন্স বা অন্য কোন যানবাহন ব্যবহারের সুযোগ আছে ? (যা এই স্বাস্থ্যসেবাকেন্দ্রে সার্বক্ষণিক থাকে বা এখানে ব্যবহৃত হয়)]	Yes (হ্যাঁ) ………………………………….. 1<br>No (না) …………………………………… 2		☐
		Source (উৎস) ………………………….....		
13.	Does your facility have electricity? [আপনার স্বাস্থ্যসেবাকেন্দ্রটিতে বিদ্যুৎ সরবরাহ আছে কি?]	Yes (হ্যাঁ) ………………………………….. 1<br>No (না) …………………………………… 2		☐

	13.1. What is the facility's main source of electricity? [স্বাস্থ্যসেবাকেন্দ্রটির বিদ্যুৎ সরবরাহের প্রধান উৎস কী?]	Central Supply of Electricity (e.g. National or community grid) (বিদ্যুতের কেন্দ্রীয় সরবরাহ ব্যবস্থা (যেমন জাতীয় বা এলাকার গ্রিড)........1<br>Generator (Fuel or Battery Operated) জেনারেটর (জ্বালানি বা ব্যাটারি চালিত) ………..…..2<br>Solar System (সৌরবিদ্যুৎ ব্যবস্থা) …………….3<br>Other (Specify)(অন্যান্য (উল্লেখ করুন)] ………99	☐
	13.2. Other than the main or primary source, does the facility have a secondary or backup source of electricity? [প্রধান উৎসের পাশাপাশি, এই স্বাস্থ্যসেবাকেন্দ্রের কি বিদ্যুতের অন্য কোন উৎস রয়েছে?]	Yes (হ্যাঁ) ……………………………….. 1<br>No (না) ………………………………… 2	☐
	13.2.1 If Yes (Specify) [যদি হ্যাঁ হয়, তবে উল্লেখ করুন]		
14.	On average, how many hours per day is this facility open? [এই স্বাস্থ্যসেবাকেন্দ্রটি গড়ে প্রতিদিন কত ঘন্টা খোলা থাকে?]	4 Hours or less (৪ ঘন্টা বা তার কম) .... 1<br>5 to 8 Hours (৫ থেকে ৮ ঘন্টা) .... 2<br>9 to 16 Hours (৯ থেকে ১৬ ঘন্টা)...... .... 3<br>17 to 23 Hours (১৭ থেকে ২৩ ঘন্টা)................ 4<br>24 Hours (২৪ ঘন্টা).................................... 5	☐
	**Services (পরিষেবা সমূহ)**		

		A) Diagnosis Availability (সনাক্তকরণ সহজলভ্যতা)		B)Management Availability (চিকিৎসা সহজলভ্যতা)		C) Service provider(s) received any training in last two year (সেবা প্রদানকারী/রা গত ২ বছরে কোন ট্রেনিং নিয়েছে কি)	
15.	Does this facility offer diagnosis or management of non-communicable diseases [এই স্বাস্থ্যসেবাকেন্দ্রে কি অসংক্রামক ব্যধি সনাক্তকরণ বা চিকিৎসা দেয়া হয় ?]	Yes (হ্যাঁ)	No (না)	Yes (হ্যাঁ)	No (না)	Yes (হ্যাঁ)	No (না)
	Diabetes (ডায়াবেটিস)	1	2	1	2	1	2
	Hypertension (উচ্চরক্তচাপ)	1	2	1	2	1	2
	Chronic respiratory diseases (দীর্ঘস্থায়ী শ্বাসকষ্টের রোগ)	1	2	1	2	1	2
	Cervical cancer (জরায়ুর ক্যান্সার)	1	2	1	2	1	2

16.	Does this facility offer diagnosis or management of acute vascular events such as STROKE, Myocardial Infarction [এই স্বাস্থ্যসেবাকেন্দ্রে কি হৃদরোগের (যেমন স্ট্রোক, মায়োকারডিয়াল ইনফার্কশন) জরুরি ভিত্তিতে সনাক্তকরণ বা চিকিৎসা দেয়া হয় ?]	Yes (হ্যাঁ) ………………………….. 1<br>No (না) …………………………… 2	☐
	If Yes (হ্যাঁ হলে)		
	16.1. What sort of management you provide? (কি ধরণের চিকিৎসা দেয়া হয়?)	Screening only [শুধুমাত্র স্ক্রিনিং] ………… 1<br>Screening & management [স্ক্রিনিং এবং চিকিৎসা] ……….. ....2<br>Screening and referral [স্ক্রিনিং এবং রেফারেল] ……………. 3	☐<br>If 3→ 16.3
	16.2. Who attend the patient first (কে প্রথম রোগীর পরিচর্যা করে)	Doctor (ডাক্তার) ………………………… 1<br>Nurse (নার্স) ……………..……….. 2<br>Medical Assistant (মেডিক্যাল অ্যাসিস্ট্যান্ট)... 3<br>Other (Specify) (অন্যান্য, উল্লেখ করুন) …… 9	
	If No (না হলে)		
	16.3. Do you refer the patient with initial screening & referral note [আপনারা কি রোগীকে প্রাথমিক স্ক্রিনিং ও রেফারেল নোট সহকারে রেফার করেন ?]	Yes (হ্যাঁ) ……………………………….. 1<br>No (না) ………………………………… 2	☐
17.	In case of referral of stroke or MI cases where do you usually refer? [স্ট্রোক বা মায়োকারডিয়াল ইনফার্কশনের ক্ষেত্রে রোগীকে আপনারা কোথায় রেফার করেন ?]	Government tertiary care facilities [সরকারী স্বাস্থ্যসেবাকেন্দ্র]...1<br>District hospital [জেলা হাসপাতাল].............2<br>Upazila health complex [উপজেলা হাসপাতাল]	☐<br>☐<br>☐

		....3<br>Privet Hospital [বেসরকারী হাসপাতাল] ….... 4<br>Other (Specify) অন্যান্য (উল্লেখ করুন) ……...9	☐ ☐
	17.1. Do you provide logistics support (e.g ambulance services, oxygen personnel etc.) for referral [রেফারেলের ক্ষেত্রে আপনারা কি কোনভাবে (অ্যাম্বুলেন্স, অক্সিজেন সরবরাহকারী ইত্যাদি দ্বারা) সহযোগিতা করেন ?]	Yes (হ্যাঁ) ……………………………….. 1<br>No (না) ………………………………… 2	☐
	17.2. Do you claim charges for referral note? [রেফারেলের নোটের ক্ষেত্রে আপনারা কি কোন টাকা নেন ?]	Yes (হ্যাঁ) ……………………………….. 1<br>No (না) ………………………………… 2	☐
18.	Do you keep record of patient with acute emergency [একুইট ইমারজেন্সি হয়েছে এমন রোগীদের কোন রেকর্ড কি আপনারা রাখেন ?]	Yes (হ্যাঁ) ……………………………….. 1<br>No (না) ………………………………… 2	☐
	18.1. How do you keep records of patients with acute vascular events? [একুইট ভাস্কুলার সমস্যায় আক্রান্ত রোগীদের রেকর্ড আপনারা কীভাবে রাখেন ?]	Maintain patient register [রোগীদের রেজিস্টার রাখা হয়] …………................................... 1<br>Electronically capture & upload [ইলেকট্রনিকভাবে রাখা হয়] ….. ....2<br>Other (Specify) [অন্যান্য (উল্লেখ করুন)] 9	☐
	18.2. How long do you preserve records? (কতদিন পর্যন্ত আপারা রেকর্ডগুলো সংরক্ষণ করেন?)	☐☐ Years [বছর] ☐☐months [মাস]	
19.	**Equipment [সরঞ্জাম]**<br>Please tell me if the following equipment and supplies used in the provision of client services are available and functional in this facility today. (দয়া করে আমাকে জানাবেন, নিম্নউল্লেখিত সরঞ্জামাদির মধ্যে কোনগুলো এই প্রতিষ্ঠানে আছে এবং কার্যকর অবস্থায় আছে)।		

		A) AVAILABLE (ব্যবহারযোগ্য)			B) Functioning (কার্যকরী)		
		Observed [দেখেছি]	Reported not seen [আছে, তবে দেখিনি]	Not Available [নেই]	Yes [হ্যাঁ]	No [না]	Don’t Know [জানি না]
a.	Weighing scale (ওজন মাপার স্কেল)	1→B	2→B	3	1	2	8
b.	Measuring tape/stadiometre (পরিমাপ করার ফিতা/ স্টেডিওমিটার)	1→B	2→B	3	1	2	8
c.	Thermometer (থার্মোমিটার)	1→B	2→B	3	1	2	8
d.	Sthethoscope (স্টেথোস্কোপ)	1→B	2→B	3	1	2	8
e.	Blood pressure apparatus (may be digital or manual sphygmomanometer with stethoscope) [রক্তচাপ পরিমাপ করার যন্ত্রপাতি (স্টেথোস্কোপ এর সাথে ডিজিটাল বা ম্যানুয়াল sphygmomanometer হতে পারে।)]	1→B	2→B	3	1	2	8
f.	Intravenous infusion kits	1→B	2→B	3	1	2	8
g.	Oxygen cylinders (অক্সিজেন সিলিন্ডার)	1→B	2→B	3	1	2	8
h.	Flowmeter for oxygen therapy (with humidification) [অক্সিজেন থেরাপি দেওয়ার জন্য Flowmeter (আদ্রতার সাথে)]	1→B	2→B	3	1	2	8
i.	Oxygen delivery apparatus (key connecting tubes and mask/nasal prongs) [শরীরে অক্সিজেন সরবরাহ করার যন্ত্রপাতি (সিলিন্ডারের সাথে সংযোগ করার নল ও মাস্ক/ নাকের কাঁটা)	1→B	2→B	3	1	2	8
j.	Glucometer (গ্লুকোজ পরিমাপের যন্ত্র)	1→B	2→B	3	1	2	8
k.	Glucometer test strips (with valid expiration date) [গ্লুকোজ পরিমাণ পরীক্ষা করার কাঁঠি (বৈধ মেয়াদপূর্তির তারিখ সহ)]	1→B	2→B	3	1	2	8

l.	ECG machine (ইসিজি মেশিন)	1→B	2→B	3	1	2	8
m.	Echo- cardiography (ইকোকার্ডিওগ্রাফি)	1→B	2→B	3	1	2	8
n.	Pulse oximetry (পালস অক্সিমেট্রি)	1→B	2→B	3	1	2	8
o.	Cardiac catheterization (কার্ডিয়াক ক্যাথেটার)	1→B	2→B	3	1	2	8
p.	CT Scan (সিটি স্ক্যান)						

19.1	At any time during the past 3 months has oxygen been unavailable for any reason? (গত ৩ মাসের যেকোনো সময়ে কোনো কারণে কি এমন হয়েছে যে অক্সিজেন ছিলো না?)	Yes (হ্যাঁ) ……………………………….. 1<br>No (না) ………………………………… 2	☐
	**Medicine & Commodities (ঔষধ প্রত্যাদি)**		
20.	Does this facility stock medicines, vaccines, or contraceptive commodities? (এই স্বাস্থ্যকেন্দ্র কি ঔষধ, টিকা অথবা গর্ভনিরোধক পন্য মজুদ করে?)	Yes (হ্যাঁ) ……………………………….. 1<br>No (না) ………………………………… 2	☐

21	Are any of the following medicines for the management of acute vascular events available in the facility today? [নিম্নউল্লেখিত ঔষধের মধ্যে একিউট ভাস্কুলার সমস্যার চিকিৎসার জন্য কোনগুলো আজ এই স্বাস্থ্যকেন্দ্রে রয়েছে ?]	Observed Available [আছে এবং দেখেছি]		Not Observed [দেখিনি]		
		At least one valid [অন্তত একটির মেয়াদ আছে]	Available non valid [আছে, তবে মেয়াদ উত্তীর্ণ]	Reported available but not seen [আছে, তবে দেখিনি]	Not available today [আজকে নেই]	Never available [কখনই ছিলো না]
a.	Aspirin	1	2	3	4	5
b.	NSAID	1	2	3	4	5
c.	Metformin	1	2	3	4	5
d.	Insulin regular injection	1	2	3	4	5
e.	ACE inhibitor (e.g. enalapril, lisinopril, ramipril, perindopril)	1	2	3	4	5
f.	Thiazide (e.g. hydrochlorothiazide)	1	2	3	4	5
g.	Beta blocker (e.g.bisoprolol, metoprolol, carvedilol, atenolol)	1	2	3	4	5
h.	Calcium channel blocker (e.g. amlodipine)	1	2	3	4	5
i.	Simvastatin tablet or other statin e.g. atorvastatin, pravastatin, fluvastatin	1	2	3	4	5
j.	Omeprazole or alternative such as pantoprazole, abeprazole	1	2	3	4	5
k.	Furosemide	1	2	3	4	5
l.	Prednisolone	1	2	3	4	5
m.	Epinephrine	1	2	3	4	5
n.	Hydrocortisone	1	2	3	4	5
o.	Streptokinase (thrombolysis)	1	2	3	4	5
p.	Glyceryl trinitrate sublingual	1	2	3	4	5

We have now completed all of the questions in this module of the survey. Thank you for your participation. [আমরা এই জরীপের সবগুলো প্রশ্নের উত্তর সম্পন্ন করেছি । এতে অংশগ্রহণ করার জন্য আপনাকে অসংখ্য ধন্যবাদ।]

Interviewer's Comments

Name: **Signature** **Date:**
