# Supplementary material for: Access to primary health care for acute vascular events in rural low income settings: a mixed methods study
Source: BMC Health Serv Res. 2017 Jan 18;17:47. doi: 10.1186/s12913-017-1987-8 (PMC5242000; doi:10.1186/s12913-017-1987-8)
Supplement: Additional file 2: — Questionnaire to identify care seeking pattern during acute vascular event. (PDF 722 kb) [file 12913_2017_1987_MOESM2_ESM.pdf]

icddr,b

Health Care seeking pattern during fatal and non-fatal acute vascular events

**IDENTIFICTION**	
**Fatal Case** **Study ID** ☐☐☐☐	**Non-Fatal Case** **Study ID** ☐☐☐☐
**Name:** ………………………………………………<br>**CID:** ☐☐☐☐☐☐☐☐☐<br>**RID:** ☐☐☐☐☐☐☐☐☐☐<br>**Date of birth:** ☐☐ (**Day**) ☐☐ (**Month**) ☐☐☐☐ (**Year**)<br>**Date of death:** ☐☐ (**Day**) ☐☐ (**Month**) ☐☐☐☐ (**Year**)<br>**Date of Interview:** ☐☐ (**Day**) ☐☐ (**Month**) ☐☐☐☐ (**Year**)	**Name:** ………………………………………………<br>**CID:** ☐☐☐☐☐☐☐☐☐<br>**RID:** ☐☐☐☐☐☐☐☐☐☐<br>**Date of birth:** ☐☐ (**Day**) ☐☐ (**Month**) ☐☐☐☐ (**Year**)<br>**Date of death:** ☐☐ (**Day**) ☐☐ (**Month**) ☐☐☐☐ (**Year**)<br>**Date of Interview:** ☐☐ (**Day**) ☐☐ (**Month**) ☐☐☐☐ (**Year**)

**The Respondent is a**	**a) Non-Fatal Stroke/MI Case** ------------------------------ 1<br>**b) Caregiver of a fatal/Non-Fatal Stroke/MI Case** ---- 2	☐

SL #	Question	Code	Result	Skip code
	**Socio-demographic Information**			
1.	What is your/patient's sex? (আপনার/ রোগীর লিঙ্গ?)	Male (পুরুষ)--------------------------- 1<br>Female (মহিলা)------------------------ 2	☐	
2.	What is your/patients marital status? (আপনার/ রোগীর বৈবাহিক অবস্থা?)	Never married (কখনও বিয়ে হয়নি)----- 1<br>Married (বিবাহিত)--------------------- 2<br>Separated/Divorced (বিচ্ছিন্ন/ স্বামী পরিত্যাক্তা)--------- 3<br>Widow/Widower (বিধবা/ বিপত্নীক)----- 4	☐	
3.	Do you/patient have any children? (আপনার/ রোগীর কোন সন্তান আছে?)	Yes (হ্যাঁ) ……………………….. 1<br>No (না) ………………………… 2	☐	If 2→Q4
	3.a Sons [ছেলে]	--------------------- Number (সংখ্যা)	☐☐	
	3.b Daughters [মেয়ে]	--------------------- Number (সংখ্যা)	☐☐	
4.	What is your religion? (আপনার ধর্ম কি?)	Islam (মুসলমান)------------------------ 1<br>Hindu (হিন্দু)--------------------------- 2<br>Christian (খ্রীষ্টান)---------------------- 3<br>Buddist (বৌদ্ধ)------------------------ 4<br>Other (Specify) (অন্যান্য, উল্লেখ করুন)-- 9	☐	
5.	What is your occupation? (আপনার পেশা কি?)			
	**1 = Unskilled manual:** (দিনমজুর, রিক্সাচালক, কৃষি শ্রমিক ইত্যাদি)<br>**2 = Skilled manual:** (টেম্পু চালক, মেকানিক, অপারেটর, কাঠ মিস্ত্রি, পুলিশ, আর্মি ইত্যাদি)<br>**3 = Non-manual:** (দোকানদার, জমির মালিক কিন্তু জমিতে কাজ করে না, বসে বসে চাকুরী করা, ব্যবসায়ী, শ্রমিক নেতা ইত্যাদি)<br>**4 = Professional=** (ডাক্তার, প্রকৌশলী, শিক্ষক ইত্যাদি)	Unskilled manual [অদক্ষ কায়িক] ....1<br>Skilled manual [দক্ষ কায়িক] ....2<br>Non manual [কায়িক নয়] ....3<br>Professional [পেশাদার] ....4<br>Housewife [গৃহিনী] ....5<br>Unemployed [বেকার] ....6<br>Other (Specify) (অন্যান্য, উল্লেখ করুন).....9	☐	

SL #	Question	Code	Result	Skip code
6.	Who is the head of your/patient family? [আপনার /রোগীর পরিবারের প্রধান কে?]	Self, Male [পুরুষ, অংশগ্রহণকারী নিজে] ----- 1<br>Self, Female [মহিলা, অংশগ্রহণকারী নিজে]-- 2<br>Else, Male [অন্য পুরুষ] ------------------ 3<br>Else, Female [অন্য মহিলা]---------------- 4	☐	
7.	What is the main occupation of the household head? [পরিবার প্রধানের পেশা কি?]			
	**1 = Unskilled manual:** (দিনমজুর, রিক্সাচালক, কৃষি শ্রমিক ইত্যাদি)<br>**2 = Skilled manual:** (টেম্পু চালক, মেকানিক, অপারেটর, কাঠ মিস্ত্রি, পুলিশ, আর্মি ইত্যাদি)<br>**3 = Non-manual:** (দোকানদার, জমির মালিক কিন্তু জমিতে কাজ করে না, বসে বসে চাকুরী করা, ব্যবসায়ী, শ্রমিক নেতা ইত্যাদি)<br>**4 = Professional=** (ডাক্তার, প্রকৌশলী, শিক্ষক ইত্যাদি)	Unskilled manual [অদক্ষ কায়িক] ....1<br>Skilled manual [দক্ষ কায়িক] ....2<br>Non manual [কায়িক নয়] ....3<br>Professional [পেশাদার] ....4<br>Housewife [গৃহিনী] ....5<br>Unemployed [বেকার] ....6<br>Other (Specify) (অন্যান্য, উল্লেখ করুন).....9	☐	
8.	Household average monthly income during the last year? গত বছর পরিবারের (খানা) গড় মাসিক আয় কত ছিল?	-------------------- Taka/month [টাকা/প্রতি মাসে]		
9.	What is the education qualification of you/patient? [আপনার /রোগীর শিক্ষাগত যোগ্যতা কী ?]	Never went to school<br>[কখনো স্কুলে যাইনি]--------------------------1<br>Primary (Below class 5)<br>[৫ম শ্রেণীর আগ পর্যন্ত] ---------------------2<br>Primary (Passed class 5)<br>[৫ম শ্রেণী পাশ]----------------------------3<br>Secondary (Class 6-10/Passed)<br>[মাধ্যমিক (৬ষ্ঠ থেকে ১০ম/পাশ)] ------------ 4<br>Higher secondary (Class twelve /Passed) [উচ্চ মাধ্যমিক (১২ তম শ্রেণী/পাশ)]<br>-- 5<br>Higher education [উচ্চ শিক্ষা]------------ 6<br>Others (specify) [(অন্যান্য, উল্লেখ করুন)]-- 9	☐	
	**History of Previous Illness (পূর্ব অসুস্থতার বিবরণ)**			
10.	Do you/patient had the history of following illness [আপনার/রোগীর কি নিচের রোগগুলোর কোনটি ছিলো?]	Yes (হ্যাঁ) ………………………….. 1<br>No (না) …………………………… 2		
a.	Angina/Chest Pain (এনজাইনা/বুকে ব্যাথা)		☐	
b.	Prior myocardial infarction (মায়োকার্ডিয়াল ইনফার্কশন)		☐	
c.	Diabetes (ডায়াবেটিস)		☐	
d.	Hypertension (উচ্চ রক্তচাপ)		☐	
e.	Heart Failure (হার্ট ফেল)		☐	
f.	Hyperlipidemia (রক্তে চর্বির পরিমান বেড়ে যাওয়া)		☐	
g.	Prior stroke or transient ischemic attack (পূর্বের স্ট্রোক)		☐	

SL #	Question	Code	Result	Skip code
	**Risk Factors (ঝুঁকির কারণসমূহ)**			
11.	**Smoking** *[ধূমপান] :*<br>Now I am going to ask your/patient's smoking (cigarette, beeri, etc.) habit. Answer these questions if you/patient are a current smoker or if you/patient have ever smoked in the past:<br>[এখন আমি আপনাকে ধূমপান বিষয়ক (যেমন- সিগারেট, বিড়ি ইত্যাদি সম্পর্কে) কিছু প্রশ্ন করব। যদি আপনি /রোগী বর্তমানে ধূমপায়ী হন অথবা পূর্বে ধূমপান করে থাকেন তাহলে প্রশ্নগুলির উত্তর দিন।]			

	**Type/** ধরণ	**a.** **1=Yes /হ্যাঁ** **2= No/না**	**b.** **User** /ব্যবহারকারী		**c.** **Daily** /দৈনিক	**d.** **Occasionally** /মাঝেমাঝে	**e.** **Start** /শুরু	**f.** **Quit** /ছেড়ে দেয়া	**g.** **Total Duration** /মোট সময়কাল
		If `2 = No, then skip to the next question *[যদি উত্তর '2= না হয়, তাহলে পরবর্তী প্রশ্নে চলে যান]*	**b1.** **Current** [বর্তমানে]	**b2.** **Former** [অতীতে]	**Sticks / Day** [দিনে কয়টি বা কতবার]	**Sticks/ Day** [দিনে কয়টি বা কতবার]	**Age** /বয়স	**Age** /বয়স	**Age Years** /বছর
a.	Cigarette/ সিগারেট								
b.	Beeri/ বিড়ি								
c.	Hukka/হুক্কা								
d.	Gul/ গুল								
e.	Jarda /জর্দা								
f.	Tamak/ তামাক								

SL #	Question	Code	Result	Skip code
12.	**Diet** /খাদ্যাভ্যাস			
a.	In a typical week, on how many days do you/patient eat fruit? [সচরাচর সপ্তাহের কতদিন আপনি/রোগী ফল খান/খেতেন ?]	|__|__| number of days/ দিন<br>Don't Know /জানি না 77	☐☐	
b.	How many servings of fruit do you/patient eat on one of those days? [সেসব দিনে মোট কতবার আপনি/রোগী ফল খান/খেতেন?]	|__|__| number of servings / টি পরিবেশন	☐☐	
c.	In a typical week, on how many days do you eat vegetables? [সচরাচর সপ্তাহের কতদিন আপনি/রোগী শাক-সবজি খান/খেতেন ?]	|__|__| number of days/ দিন<br>Don't Know /জানি না 77	☐☐	
d.	How many servings of vegetables do you/patient eat on one of those days? [সেসব দিনে মোট কতবার আপনি/রোগী শাক-সবজির খান/খেতেন ?]	|__|__| number of servings/ টি পরিবেশন	☐☐	
e.	How often do you/patient add salt in the foods right before eating? [সচরাচর খাবার খাওয়ার ঠিক আগ মুহূর্তে আপনি/রোগী কি কখনো পাতে আলগা লবণ নেন/নিতেন ?]	Always সবসময় ------------------------- 1<br>Often প্রায়শই ----------------------------- 2<br>Sometime কখনো কখনো ----------------- 3<br>Rarely খুবই কম সময়--------------------- 4<br>Never কখনই না -------------------------- 5<br>Don't know জানি না -------------------- 7	☐	
f.	How often do you/patient added salt in cooking or preparing foods in the household? [আপনার/রোগী খাবার রান্না/তৈরি করার সময় লবণ ব্যবহার করা হয়?]	Always সবসময় ------------------------- 1<br>Often প্রায়শই ----------------------------- 2<br>Sometime কখনো কখনো ----------------- 3<br>Rarely খুবই কম সময়--------------------- 4<br>Never কখনই না -------------------------- 5<br>Don't know জানি না -------------------- 7	☐	

SL #	Question	Code	Result	Skip code
g.	How much salt do you think you/patient consume? [আপনি/রোগী কতটুকু লবণ খান বলে আপনি মনে করেন?]	Far too much অনেক অনেক বেশী ---------- 1<br>Too much অনেক বেশী--------------------- 2<br>Just the right amount [ঠিক পরিমাণমতো]<br>-- 3<br>Too little খুব কম------------------------ 4<br>Far too little অতিরিক্ত কম---------------- 5<br>Don't know জানি না -------------------- 7	☐	
	**History of raised Blood pressure & Diabetes/ উচ্চ রক্তচাপ এবং ডায়েবিটিস সংক্রান্ত তথ্য**			
13.	Have you/patient ever had your/patient blood pressure measured by a doctor or other health worker? আপনি/রোগী কি কখনো ডাক্তার বা অন্য স্বাস্থ্যকর্মীর কাছে রক্তচাপ পরীক্ষা করিয়েছেন ?	Yes (হ্যাঁ) ………………………….. 1<br>No (না) …………………………… 2	☐	
	13.a. If Yes who measure (যদি হ্যাঁ হয় তবে কে পরিমাপ করেছিলেন)	______________________		
14.	Have you/patient ever had your/patient blood pressure measured by a doctor or other health worker before stroke/MI? স্ট্রোক বা এম.আই হবার আগে কি আপনি/রোগী একজন ডাক্তার বা অন্য স্বাস্থ্যকর্মীর কাছে রক্তচাপ পরীক্ষা করিয়েছিলেন ?	Yes (হ্যাঁ) ………………………….. 1<br>No (না) …………………………… 2	☐	
	14.a. If Yes who measure (যদি হ্যাঁ হয় তবে কে পরিমাপ করেছিলেন)	______________________		
15.	Have you/patient ever been told by a doctor or other health worker that You/patient have raised blood pressure/hypertension? আপনাকে/রোগীকে কি কখনো কোন ডাক্তার বা অন্য স্বাস্থ্যকর্মী বলেছেন যে উচ্চ রক্তচাপ আছে ?	Yes (হ্যাঁ) ………………………….. 1<br>No (না) …………………………… 2	☐	
	15.a. If Yes who told (যদি হ্যাঁ হয় তবে কে বলেছিলেন)	______________________		
16.	Are you currently /was the patient taking any medication for hypertension? আপনি/রোগী কি উচ্চ রক্তচাপ নিয়ন্ত্রণের কোন ঔষধ খাচ্ছেন/খেতেন?	Yes (হ্যাঁ) ………………………….. 1<br>No (না) …………………………… 2	☐	
17.	Have you/patient ever had your/ patient blood sugar measured by a doctor or other health worker? আপনি/রোগী কি কখনো ডাক্তার বা অন্য স্বাস্থ্যকর্মীর কাছে রক্তে চিনি/শর্করার (ডায়াবেটিস))পরিমাণ পরীক্ষা করিয়েছেন/ করিয়েছিলেন?	Yes (হ্যাঁ) ………………………….. 1<br>No (না) …………………………… 2	☐	
	17.a. If Yes who measure (যদি হ্যাঁ হয় তবে কে পরিমাপ করেছিলেন)	______________________		
18.	Have you/patient ever had your/ patient blood sugar measured by a doctor or other health worker before stroke/MI? স্ট্রোক বা এম.আই হবার আগে কি আপনি/রোগী একজন ডাক্তার বা অন্য স্বাস্থ্যকর্মীর কাছে রক্তে চিনি/শর্করার পরিমাণ পরীক্ষা করিয়েছিলেন?	Yes (হ্যাঁ) ………………………….. 1<br>No (না) …………………………… 2	☐	
	18.a. If Yes who measure (যদি হ্যাঁ হয় তবে কে পরিমাপ করেছিলেন)	______________________		

SL #	Question	Code	Result	Skip code
19.	Have you/patient ever been told by a doctor or other health worker that You/patient have Diabetes? আপনাকে/রোগীকে কি কখনো কোন ডাক্তার বা অন্য স্বাস্থ্যকর্মী বলেছেন যে ডায়েবিটিস আছে ?	Yes (হ্যাঁ) …………………………….. 1<br>No (না) …………………………… 2	☐	
	19.a. If Yes who told (যদি হ্যাঁ হয় তবে কে বলেছিলেন)	________________________________		
20.	Are you currently /was the patient taking any medication for diabetes? আপনি/রোগী কি ডায়েবিটিস নিয়ন্ত্রণের কোন ঔষধ খাচ্ছেন/খেতেন?	Yes (হ্যাঁ) …………………………….. 1<br>No (না) …………………………… 2	☐	
	**Care Seeking/ স্বাস্থ্যসেবা গ্রহণ সংক্রান্ত তথ্য**			
21.	When did you/patient get stroke/MI আপনার/রোগীর স্ট্রোক/এম.আই কবে হয়েছিলো?	☐☐ ☐☐ ☐☐☐☐<br>**Day** **Month** **Year**<br>দিন মাস বছর		
	21.1. Time of attack অ্যাটাক কখন হয়েছিলো?	|__|__|.|__|__| am/pm		
22.	When you/patient had stroke was you/h/she conscious? যখন স্ট্রোক/এম.আই হয়েছিলো তখন কি আপনি/রোগী চেতন অবস্থায় ছিলেন?	Yes (হ্যাঁ) ---------------------------- 1<br>No (না) ------------------------------ 2<br>Don’t know (জানিনা) ---------------- 7	☐	
23.	When you/patient had stroke/MI were you living alone? যখন স্ট্রোক/এম.আই হয়েছিলো তখন কি আপনি/রোগী বাসায় একা ছিলেন?	Yes (হ্যাঁ) …………………………….. 1<br>No (না) …………………………… 2	☐	
24.	Did you/patient or any other family member recognize the symptoms of stroke/MI? স্ট্রোক/এম.আই হয়েছে এমন লক্ষণগুলো কি আপনি/রোগী বা পরিবারের অন্য কেউ বুঝতে পেরেছিলেন?	Yes (হ্যাঁ) ---------------------------- 1<br>No (না) ------------------------------ 2<br>Don’t know (জানিনা) ---------------- 7	☐	
25.	Did any household member/relative have stroke/MI before your/patient attack? আপনার/রোগীর আগে পরিবারের অন্য কারো কি স্ট্রোক/এম.আই হয়েছিলো?	Yes (হ্যাঁ) …………………………….. 1<br>No (না) …………………………… 2	☐	
26.	When the attack happen do you/patient/any HH member consult with anyone to seek medical attention? যখন আপনার/রোগীর স্ট্রোক/এম.আই হলো তখন কি পরিবারের কেউ চিকিৎসার ব্যাপারে কারো সাথে পরামর্শ করেছিলেন ?	Yes (হ্যাঁ) …………………………….. 1<br>No (না) …………………………… 2	☐	If Yes go to 26.a<br>If no go to 26.b
	26.a. If yes, Whom did you consult? যদি হ্যাঁ হয়, তবে কার সাথে পরামর্শ করেছিলেন ?	Physician ডাক্তার ---------------------- 1<br>Any health care provider<br>কোন স্বাস্থ্যসেবা প্রদানকারী ---------------- 2<br>Any family member<br>পরিবারের কোন সদস্য --------------------- 3<br>Neighbour/nonrelatives প্রতিবেশী/আত্মীয় নয় এমন------------------------ 4	☐	
	26.b. If no, why? যদি না হয়, তবে কেন ?	Appraisal of symptoms as not being serious or urgent<br>[লক্ষণগুলো গুরুতর মনে হয়নি] ---------------- 1<br>Wait for symptoms to go away<br>[লক্ষণগুলো এমনি চলে যাবে বলে অপেক্ষা] ----- 2<br>Concern about troubling other [অন্যকে ঝামেলায় ফেলার উদ্বেগ] -------------------- 3<br>Other (Specify) অন্যান্য (উল্লেখ করুন)-----9	☐	

SL #	Question	Code	Result	Skip code
27.	How long it took to make a decision to seek medical attention after the attack? অ্যাটাকের কতক্ষণ পর মেডিকাল চিকিৎসা গ্রহণের সিদ্ধান্ত নেয়া হয়েছিলো ?	|__|__| hour ঘন্টা |__|__| minute মিনিট		
	27.a. Did he/she die at home before seeking medical attention? (ask only for fatal stroke/MI cases) (শুধুমাত্র প্রাণনাশক স্ট্রোক/এম.আই-এর ক্ষেত্রে) রোগী কি কোন ধরনের চিকিৎসা নেবার আগেই বাড়িতেই মারা গিয়েছিলো?	Yes (হ্যাঁ) ………………………….. 1<br>No (না) …………………………… 2	☐	
28.	What kind of medical contact did you/patient made? আপনি/রোগী কী ধরণের চিকিৎসা নিয়েছিলেন?	Took the patient to nearest medical facilities রোগীকে নিকটবর্তী স্বাস্থ্যসেবাকেন্দ্রে নিয়ে যাওয়া ---------------------------- 1<br>Took the patient directly to specialist hospital রোগীকে সরাসরি বিশেষায়িত হাসপাতালে নিয়ে যাওয়া ----------------------------- 2<br>Bring a qualified physician at home অভিজ্ঞ ডাক্তারকে বাসায় নিয়ে আসা -------------------------------- 3<br>Bring any other health care provider at home অন্য কোন স্বাস্থ্যসেবা প্রদানকারীকে বাসায় নিয়ে আসা ------------------------- 4<br>No medical contact (কোন চিকিৎসা বা স্বাস্থ্যসেবা গ্রহন করা হয়নি)---------------- 5	☐	
	28a. What was the reason for not taking any medical contact (কোন চিকিৎসা বা স্বাস্থ্যসেবা গ্রহন না করার কারন কি ছিল?)			
29.	How long it took to make first medical contact after the attack? অ্যাটাক হবার পর প্রথম মেডিক্যাল চিকিৎসা পেতে কতক্ষণ সময় লেগেছিলো ?	|__|__| hour ঘন্টা |__|__| minute মিনিট		
	29.1. Did he/she die in-between decision to seek care and first medical contact and? (ask only for fatal stroke/MI cases) (শুধুমাত্র প্রাণনাশক স্ট্রোক/এম.আই-এর ক্ষেত্রে) চিকিৎসা নেবার সিদ্ধান্ত গ্রহণ এবং চিকিৎসা পাওয়ার মধ্যবর্তী সময়ে কি রোগী মারা গিয়েছিলো?	Yes (হ্যাঁ) ………………………….. 1<br>No (না) …………………………… 2	☐	
30.	What mode of transport did you/patient used to reach fist medical contact? (if qualified provider/other healthcare worker brought at home what mode of transport was used for him/her transport) আপনাকে/রোগীকে চিকিৎসার জন্য নিয়ে যেতে, বা ডাক্তার/স্বাস্থ্যসেবীকে বাসায় নিয়ে আসতে কোন যানবাহন ব্যবহৃত হয়েছিলো ?	Ambulance অ্যাম্বুলেন্স -------------------- 1<br>Rickshaw/VAN রিকশা/ভ্যান ------------ 2<br>CNG auto rickshaw সি.এন.জি অটো রিকশা ---------------- 3<br>Other অন্যান্য ---------------------------- 9	☐	
	30.1. Cost of Transpiration<br>যাতায়াত বাবদ খরচ	-------------------- Taka/month টাকা/প্রতি মাসে		
	[যদি প্রশ্ন ২৮-এর উত্তর ৩ বা ৪ হয়→৩১] [যদি প্রশ্ন ২৮-এর উত্তর ১ বা ২ হয় তাহলে →৩৮]			
31.	Was qualified provider/other healthcare worker give any treatment at home? দক্ষ স্বাস্থ্যসেবাকর্মী কি বাসায় এসে কোন চিকিৎসা দিয়েছিলেন ?	Yes (হ্যাঁ) ………………………….. 1<br>No (না) …………………………… 2	☐	

SL #	Question	Code	Result	Skip code
32.	How long it took to initiate treatment at home after the attack? অ্যাটাকের কতক্ষণ পর চিকিৎসা শুরু করা হয়েছিলো ?	|__|__| hour ঘন্টা |__|__| minute মিনিট		
33.	What kind of treatment provided at home? বাসায় কী ধরণের চিকিৎসা দেয়া হয়েছিলো ?	Medication ঔষধ ------------------------ 1<br>Injection/IV infusion ইঞ্জেকশন--------- 2<br>Other (Specify) অন্যান্য (উল্লেখ করুন) ----------------------------------- 9		
34.	Were you/patient referred by the provider/other healthcare worker to any medical facilities from home? সেই স্বাস্থ্যসেবাকর্মী কি আপনাকে/রোগীকে কোন স্বাস্থ্যসেবাকেন্দ্রে যাবার জন্য রেফার করেছিলেন?	Yes (হ্যাঁ) ………………………….. 1<br>No (না) …………………………… 2	☐	If NO then move to Q.37
35.	What kind of facility he/she referred? কোন ধরণের স্বাস্থ্যসেবাকেন্দ্রে তিনি রেফার করেছিলেন?	Upazila Health complex উপজেলা স্বাস্থ্য কমপ্লেক্স ------------------------------- 1<br>District hospital জেলা হাসপাতাল ------------------- 2<br>Medical college/tertiary care facilities মেডিকেল কলেজ/টারশিয়ারি সেবাকেন্দ্র -------------------- 3<br>Private facilities বেসরকারী স্বাস্থ্যসেবাকেন্দ্র -------------------- 4<br>Other অন্যান্য ----------------------------- 9	☐	
36.	Name of the facility স্বাস্থ্যসেবাকেন্দ্রের নাম	____________________________		
37.	Cost of Treatment at home (inclusive of provider fee/medication charge etc.) বাসায় চিকিৎসা নেওয়া বাবদ খরচ (স্বাস্থ্যসেবাকর্মীর ফী, ঔষধ ইত্যাদি সহ)	-------------------- Taka/month টাকা/প্রতি মাসে		
	**[যদি প্রশ্ন ২৮-এর উত্তর ১ বা ২ হয়→৩৮]**			
38.	How long it took to hospital arrival after the attack? অ্যাটাকের পর হাসপাতাল পৌঁছাতে কত সময় লেগেছিলো?	|__|__| hour ঘন্টা |__|__| minute মিনিট		
	38.1. Did he/she die during transportation to the health facility? (ask only for fatal stroke/MI cases) (শুধুমাত্র প্রাণনাশক স্ট্রোক/এম.আই-এর ক্ষেত্রে) স্বাস্থ্যসেবাকেন্দ্রে যাবার পথেই কি রোগী মারা গিয়েছিলো?	Yes (হ্যাঁ) ………………………….. 1<br>No (না) …………………………… 2	☐	
39.	What kind of facility was that? সেটি কোন ধরণের স্বাস্থ্যসেবাকেন্দ্র ছিলো?	Upazila Health complex উপজেলা স্বাস্থ্য কমপ্লেক্স ------------------------------- 1<br>District hospital জেলা হাসপাতাল ------------------- 2<br>Medical/tertiary care facilities মেডিকেল টারশিয়ারি সেবাকেন্দ্র -------------------- 3<br>Private facilities বেসরকারী স্বাস্থ্যসেবাকেন্দ্র -------------------- 4<br>Other অন্যান্য ----------------------------- 9	☐	
40.	What kind of service you/patient received at the facilities hospital? হাসপাতালে আপনি/রোগী কোন ধরণের সেবা পেয়েছিলেন ?	Screening……………………………….1<br>Admission…………………….............2<br>Initial Management…………………….3<br>Complete treatment…………………....4<br>Referred……………………………….5	☐ ☐ ☐	

SL #	Question	Code	Result	Skip code
		Declared dead with initial screening…..6<br>Sent home…………………………..…7	☐<br>☐	
41.	What kind of treatment provided at first facility? প্রথম যেই স্বাস্থ্যসেবাকেন্দ্রে গিয়েছিলেন তাতে কোন ধরণের চিকিৎসা পেয়েছিলেন?	Medication ঔষধ ------------------------ 1<br>Injection/IV infusion ইঞ্জেকশন--------- 2<br>Screening only শুধুমাত্র স্ক্রিনিং ------------ 3<br>Other (Specify) অন্যান্য (উল্লেখ করুন)----- 9	☐	
	Please write the treatment/referral note (If available)<br>দয়া করে চিকিৎসা এবং রেফারেল নোটটি লিখুন (যদি থাকে)			
42.	How long it took to initiate treatment at the first facility after attack? অ্যাটাকের পর প্রথম স্বাস্থ্যসেবাকেন্দ্রে চিকিৎসা শুরু করতে কত সময় লেগেছিলো ?	|__|__| hour ঘন্টা |__|__| minute মিনিট		
43.	Cost of Treatment at first facility (inclusive of user fee/medication cost charge etc.) প্রথম স্বাস্থ্যসেবাকেন্দ্রে চিকিৎসা বাবদ খরচ (ভর্তি ফী/ ঔষধ ইত্যাদি সহ)	-------------------- Taka/month টাকা/প্রতি মাসে		
	**[If Q.40 is 5→44] [If Q.40 is 6/7 →Q.50]**			
44.	How did you/Patient transfered to the referred facilities রোগীকে যেখানে রেফার করা হয়েছিলো সেখানে কীভাবে স্থানান্তর করা হয়েছিলো ?	Ambulance অ্যাম্বুলেন্স -------------------- 1<br>Rickshaw/VAN রিকশা/ভ্যান ------------ 2<br>CNG auto rickshaw সি.এন.জি অটো রিকশা ---------------- 3<br>Other অন্যান্য ---------------------------- 9	☐	
45.	How long it took to the referred facility? রেফারকৃত স্বাস্থ্যকেন্দ্রে নিয়ে যেতে কত সময় লেগেছিলো ?	|__|__| hour ঘন্টা |__|__| minute মিনিট		
46.	46.1 Did he/she die during transportation to the referred facility? (ask only for fatal stroke/MI cases) (শুধুমাত্র প্রাণনাশক স্ট্রোক/এম.আই-এর ক্ষেত্রে) রেফারকৃত স্বাস্থ্যসেবাকেন্দ্রে যাবার পথেই কি রোগী মারা গিয়েছিলো?	Yes (হ্যাঁ) ………………………….. 1<br>No (না) …………………………… 2	☐	
47.	How long it took to initiate treatment at the facility after attack? অ্যাটাকের পর রেফারকৃত স্বাস্থ্যসেবাকেন্দ্রে চিকিৎসা শুরু করতে কত সময় লেগেছিলো?	|__|__| hour ঘন্টা |__|__| minute মিনিট		
48.	Please write the detail treatment history (Please review medical record if available)<br>দয়া করে চিকিৎসার বিস্তারিত বিবরণ লিখুন (যদি মেডিকাল রেকর্ড থাকে তবে তা পড়ে লিখুন)			

SL #	Question	Code	Result	Skip code
49.	Cost of Treatment for acute emergency management (inclusive of user fee/medication cost charge etc.) একিউট ইমারজেন্সির চিকিৎসা বাবদ খরচ (ভর্তি ফী/ ঔষধ ইত্যাদি সহ)	-------------------- Taka/month টাকা/প্রতি মাসে		
50.	Description of care seeking after stroke & MI (স্ট্রোক/এম.আই এর পর স্বাস্থ্যসেবা গ্রহনের বর্ণনা)			

	Name of the facility (স্বাস্থ্যসেবা প্রতিষ্ঠান/সেবাদানকারীর নাম)	Type of services takes (সেবার ধরন)<br>1=Inpatient (ভর্তি)<br>2=Outpatient (বর্হিবিভাগ)	Treatment Description (চিকিৎসার বর্ণনা)	Duration of treatment (সময়)

[If Q.40=6, or 46=1 then Questionnaire is done at this point]

SL #	Question	Code	Result	Skip code
51.	Are you currently taking any medication (only for non fatal stroke/MI)? আপনি কি বর্তমানে কোন ঔষধ সেবন করছেন (শুধুমাত্র জীবতি প্রাণনাশক স্ট্রোক/এম.আই-এর ক্ষেত্রে)?	Yes (হ্যাঁ) ………………………….. 1<br>No (না) …………………………… 2	☐	

If yes, please write down the name of medicines যদি হ্যাঁ হয়, তবে ঔষধগুলোর নাম নিচে লিখুন

	Medication name ঔষধের নাম	Power পাওয়ার	Formulation Tablet ট্যাবলেট =1 Capsule ক্যাপস্যুল = 2 Injection ইঞ্জেকশন =3	Times/ day দিনে কয়বার	Numbers/ day দিনে কতটি	Regular medication নিয়মিত সেবন করেন	
						Yes =1 হ্যাঁ=১	No = 2 না=২
51.1							
51.2							
51.3							
51.4							
51.5							
51.6							
51.7							
51.8							

Thank you for your participation.
অংশগ্রহণ করার জন্য আপনাকে অসংখ্য ধন্যবাদ।

Interviewer's Comments

Name: **Signature** **Date:**
