# Supplementary material for: Access to primary health care for acute vascular events in rural low income settings: a mixed methods study
Source: BMC Health Serv Res. 2017 Jan 18;17:47. doi: 10.1186/s12913-017-1987-8 (PMC5242000; doi:10.1186/s12913-017-1987-8)
Supplement: Additional file 3: — Qualitative interview guideline. (PDF 238 kb) [file 12913_2017_1987_MOESM3_ESM.pdf]

Qualitative interview guidelines

In-depth Interview Guideline for the survivors and caregivers of Stroke or MI:

১. During Event / অ্যাটাক হবার সময়

   a. History of the acute vascular events /একিউট ভাস্কুলার ইভেন্ট সংক্রান্ড় তথ্য
   b. Recognization of symptoms/ লক্ষণগুলো চিনতে পারা
   c. First medical contact / প্রথম মেডিকাল চিকিৎসা নেয়া
   d. Perception of necessities of care seeking/ চিকিৎসা গ্রহণের ব্যপারে দৃষ্টিভঙ্গি/মতামত
   e. Decision maker of care seeking/ চিকিৎসা গ্রহণের সিদ্ধান্ড় যিনি নেন
   f. Transportation use to seek care/ চিকিৎসা নিতে যাবার যানবাহন ব্যবস্থা

2. Care seeking behaviour behaviour/ চিকিৎসা গ্রহণের ধরণ

   a. Facilities visited/ যেই যেই স্বাস্থ্যসেবাকেন্দ্রে গিয়েছেন
   b. Treatment received/ যা চিকিৎসা পেয়েছেন
   c. Any obstruction encountered during care seeking / চিকিৎসা পেতে যা যা অসুবিধা হয়েছিলো

      - Delay in seeking care/ চিকিৎসা নিতে দেরী হওয়া
      - Financial constrains/ আর্থিক সংকট
      - Transport problem/ যাতায়াতের অসুবিধা
      - Delay in starting the management/ চিকিৎসা শুর“তে দেরী
      - Lack of institutional support/ প্রাতিষ্ঠানিক সহায়তার অভাব
      - Absence of following up/ ফলো-আপের অভাব

   d. Any financial constrain/ কোনরকম আর্থিক সংকট

3. Post Events / অ্যাটাক পরবর্তী সময়ে

   a. Life after the event/ এরপরের জীবন
   b. Current treatment/ বর্তমানে যা যা চিকিৎসা পাচ্ছেন
   c. Lifestyle changes/ জীবনযাপনে যা যা পরিবর্তন এসেছে
   d. Coping with financial and other needs needs/ আর্থিক বা অন্যান্য চাহিদা যেভাবে মেটানো হচ্ছে

4. Barriers to access care/ চিকিৎসা পেতে বাধা

   a. Personal Barrier/ ব্যক্তিগত বাধা
      - Perceived need/ প্রয়োজন আছে কি না সে সম্পর্কে অভিমত
      - Quality of care/ চিকিৎসা সেবার মান
      - Perception of adequacy of the services and staff at PHC/ প্রাথমিক স্বাস্থ্যসেবাকেন্দ্রে সেবা ও কর্মচারীর পর্যাপ্ততা সম্পর্কে মতামত

- Accessibility issues including distance transport, proximity of the PHC etc./ প্রাথমিক স্বাস্থ্যসেবাকেন্দ্রে পৌঁছাতে যা যা সমস্যা (দূরত্ব, যানবাহন ইত্যাদি সহ)

b. Financial Barriers/ আর্থিক সমস্যা
   - Fee of service/ চিকিৎসা সেবার দাম
   - Out of pocket expenditure / নিজের খরচে সেবা নেওয়া
   - Charges for other services/ অন্যান্য সেবা পেতে খরচ

5. Factors that may affect utilization (Facilitators & Barriers)/ চিকিৎসা সেবা নিবার ক্ষেত্রে যেই বিষয়গুলো প্রভাব ফেলে (সুবিধা এবং অসুবিধা)

For the High level official/managers/policy makers:

1. Current services available for managing acute vascular events (Stroke and MI) at the primary care level / বর্তমানে প্রাথমিক স্বাস্থ্যসেবা কেন্দ্রগুলোতে একিউট ভাস্কুলার ইভেন্টের (স্ট্রোক/এম.আই) চিকিৎসার জন্য যা যা সেবা রয়েছে

   - Skilled Human Resource/ দক্ষ কর্মী
   - Necessary/ appropriate drugs প্রয়োজনীয় ঔষধপত্র
   - Equipments / সরঞ্জামাদি
   - Logistics

2. Adequacy of the service to cover rural population / গ্রামীন জনগোষ্ঠীর স্বাস্থ্যসেবা দেয়ার ক্ষেত্রে এই সেবাগুলো পর্যাপ্ত কি না

৩. Barriers from providers perspective / স্বাস্থ্যসেবা প্রদানকারীর মতে কি কি বাধা আছে

   - Skilled human resource / দক্ষ কর্মী
   - Guidelines for treatment / চিকিৎসার জন্য গাইডলাইন
   - Infrastructures (logistics, equipments) / অবকাঠামো
   - Supply of essential drugs/ প্রয়োজনীয় ঔষধ সরবরাহ
   - Community awareness about the service availability/ সেবা সম্পর্কে জনগণের সচেতনতার অভাব
   - Financial constrain of the patients/ রোগীদের আর্থিক সংকট

4. Action required to strengthen the existing primary care facilities for acute vascular events / প্রাথমিক স্বাস্থ্যসেবা কেন্দ্রগুলোতে একিউট ভাস্কুলার ইভেন্টের চিকিৎসা জোরদার করতে কি কি পদক্ষেপ নেয়া দরকার

   - From government level/ সরকারী পর্যায়ে
   - From NGO's and private level/ এনজিও এবং বেসরকারী পর্যায়ে
   - From community level/ এলাকায় বা সামাজিকভাবে
