# Supplementary material for: Access to primary health care for acute vascular events in rural low income settings: a mixed methods study
Source: BMC Health Serv Res. 2017 Jan 18;17:47. doi: 10.1186/s12913-017-1987-8 (PMC5242000; doi:10.1186/s12913-017-1987-8)
Supplement: Additional file 4: — Consent forms. (DOCX 21 kb) [file 12913_2017_1987_MOESM4_ESM.docx]

#
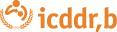


**Informed written Consent Form-English**

**(Facility Survey-Key Informant)**

| Protocol No. PR-15081 | Version No. 2.00 | Date: 13-07-2015 |
| --- | --- | --- |
| **Principal Investigator:** Shyfuddin Ahmed | | |

**Purpose of the research**

Assalamu Alaikum. Greetings from icddr,b. We came from Matlab health research facilities of icddr,b. You might know that cardiovascular disease (CVD) is globally the leading cause of morbidity and mortality. The prevalence has reduced in high income countries. It is rapidly increasing in South Asian and other low and middle income countries due to a rapid epidemiologic transition resulting in high rates of risk factors. The high burden of CVD in Bangladesh is confirmed in a recent study which found that ischemic heart disease (IHD) and stroke are top two causes of years of life lost (YLLs) in Bangladesh. On the other hand, all the common behavioral, metabolic and physiological risk factors for CVD are highly prevalent in Bangladesh. To tackle this rising epidemic of CVD health facilities in low-income and middle-income countries need to be strengthened enough to provide acute management of CVD which will prevent premature deaths and disabilities. Chronic noncommunicable disease unit (CNCDU), icddr,b is going to implement a study to find out opportunities for expanding service coverage for acute vascular events at primary care level in rural Bangladesh.

**Why selected**

We are conducting a research to explore currently available services for acute management of stroke/MI cases in the health facilities of your area. This will help us to enable current scenario of managing acute CVD events at primary level. As you are a key personal of this facility, you are being considered as recourse person for this study. Your valuable insight will help to achieve study goal, so we are inviting you to participate in this study.

**What is expected from the participants?**

If you agree to participate in this study, besides interviewing you on key issues related to emergency cardiovascular care, we will visit your health facility for observation of key items. Service-specific readiness of your health facilities for acute cardiovascular care will be explored which include type of services (emergency, curative and preventive), presence of staff and their expertise (primary care physicians and specialists), equipment and testing facilities (ECG, Echocardiography etc), availability and use of drugs (aspirin, β-blockers, calcium-channel blockers, long acting nitrates, anticoagulants etc), availability of invasive procedures (Anticoagulent treatment, Thrombolysis etc) and provision for transfer/referral (ambulance services) etc. The interview might take your 45-60 minutes including observation of key items.

**Risk and benefits**

Participation in this study virtually involves no risks. You may or may not directly be benefited from participation in the study. But your contribution will enrich our knowledge of health systems especially for service delivery of acute management of CVD for rural population. Thus your information will help to improve health care system for providing emergency cardiovascular care in Bangladesh.

**Privacy, anonymity and confidentiality**

We would like to assure you that the information collected from you including information of your health facilities will not be passed on to anybody else. Only the researchers of this study and Ethical Review Committee (ERC) will be able to look at the information. We do hereby affirm that privacy, anonymity and confidentiality of data/information identifying you will be strictly maintained. Each of you will have a code number and this code number will be used for your identification. Your name or any information about you that could be used to identify you (find out who you are) will not be published or shared with anyone else.

**Future use of information**

The information to be collected in this study might help to gain valuable experience on developing health system interventions for preventing CVD. In case of future use of the information/data collected from this study, only anonymous or abstracted information and data may be supplied to other researchers, without any conflict with or violation of the maintenance of privacy, anonymity and confidentiality of information that identifying participants in any way.

**Right not to participate and withdraw**

Your participation is voluntary. This means that you may choose not to be in this study. You may also choose to withdraw your name from the study at any time, even after you have said that you wanted to participate in the study. You don’t have to answer any question that makes you feel uncomfortable. You may ask any question about this study and we shall be happy to answer it. For further questions you may call Shyfuddin Ahmed, principal investigator of this study over telephone number 01712877165. If you feel that you have been treated unfairly or have been hurt by joining in this study you may also call M A Salam Khan, IRB Coordinator, Research Administration at icddr,b. The phone contact number of M A Salam Khan is 9827084 Ext 3206.

Do you have any questions? Yes No

Do you agree to be in the study? Yes No

Now we invite you to participate in this study. If you agree, please put your signature or your left thumbprint in the space provided below as an indication of your participation to the study.

Thank you for your cooperation.

_________________________________________________ __________________

Signature or left thumbprint of the guardian of the participant Date

_________________________________________________________ ___________________

Signature of the witness Date

________________________________________________________ ___________________

Signature of the PI or his representative (with full name & designation) Date

#
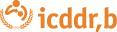


**Informed written Consent Form-English**

**(Survivor/Caregiver of Stroke or MI)**

| Protocol No. PR-15081 | Version No. 2.00 | Date: 13-07-2015 |
| --- | --- | --- |
| **Principal Investigator:** Shyfuddin Ahmed | | |

**Purpose of the research**

Assalamu Alaikum. Greetings from icddr,b. We came from Matlab health research facilities of icddr,b. You might know that cardiovascular disease (CVD) is globally the leading cause of morbidity and mortality. The prevalence has reduced in high income countries. It is rapidly increasing in South Asian and other low and middle income countries due to a rapid epidemiologic transition resulting in high rates of risk factors. The high burden of CVD in Bangladesh is confirmed in a recent study which found that ischemic heart disease (IHD) and stroke are top two causes of years of life lost (YLLs) in Bangladesh. On the other hand, all the common behavioral, metabolic and physiological risk factors for CVD are highly prevalent in Bangladesh. To tackle this rising epidemic of CVD health facilities in low-income and middle-income countries need to be strengthened enough to provide acute management of CVD which will prevent premature deaths and disabilities. Chronic noncommunicable disease unit (CNCDU), icddr,b is going to implement a study to find out opportunities for expanding service coverage for acute vascular events at primary care level in rural Bangladesh.

**Why selected**

We are conducting a research to explore health seeking patter during acute vascular event. This will help us to improve access to care for managing acute CVD events at primary level. As you are a survivor/ caregiver of stroke/MI, you can provide us valuable information to achieve our goal, so we are inviting you to participate in this study.

**What is expected from the participants?**

If you agree to participate in this study, we will ask you/your relative (who died due to stroke/MI) about your/relative’s health seeking behaviour as during his event which include information such as history of clinical symptoms, time required to reach hospital, time required for initiation of treatment, treatment that provided by in hospital, duration of the event, cost of treatment, financial hardship following event etc. The interview might take your 30-45 minutes.

**Risk and benefits**

Participation in this study virtually involves no risks. You may or may not directly be benefited from participation in the study. But your contribution will enrich our knowledge of health care service delivery for acute management of CVD for rural population.

**Privacy, anonymity and confidentiality**

We would like to assure you that the information collected from will not be passed on to anybody else. Only the researchers of this study and Ethical Review Committee (ERC) will be able to look at the information. We do hereby affirm that privacy, anonymity and confidentiality of data/information identifying you will be strictly maintained. Each of you will have a code number and this number will be used for your identification. Your name or any information about you that could be used to identify you (find out who you are) will not be published or shared with anyone else.

**Future use of information**

The information to be collected in this study might help to gain valuable experience on developing health system interventions for preventing CVD. In case of future use of the information/data collected from this study, only anonymous or abstracted information and data may be supplied to other researchers, without any conflict with or violation of the maintenance of privacy, anonymity and confidentiality of information that identifying participants in any way.

**Right not to participate and withdraw**

Your participation is voluntary. This means that you may choose not to be in this study. You may also choose to withdraw your name from the study at any time, even after you have said that you wanted to participate in the study. You don’t have to answer any question that makes you feel uncomfortable. You may ask any question about this study and we shall be happy to answer it. For further questions you may call Shyfuddin Ahmed, principal investigator of this study over telephone number 01712877165. If you feel that you have been treated unfairly or have been hurt by joining in this study you may also call M A Salam Khan, IRB Coordinator, Research Administration at icddr,b. The phone contact number of M A Salam Khan is 9827084 Ext 3206.

Do you have any questions? Yes No

Do you agree to be in the study? Yes No

Now we invite you to participate in this study. If you agree, please put your signature or your left thumbprint in the space provided below as an indication of your participation to the study.

Thank you for your cooperation.

_________________________________________________ __________________

Signature or left thumbprint of the guardian of the participant Date

_________________________________________________________ ___________________

Signature of the witness Date

________________________________________________________ ___________________

Signature of the PI or his representative (with full name & designation) Date

#
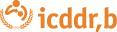


**Informed written Consent Form - English**

**(Qualitative Study)**

| Protocol No. PR-15081 | Version No. 2.00 | Date: 13-07-2015 |
| --- | --- | --- |
| **Principal Investigator:** Shyfuddin Ahmed | | |

**Purpose of the research**

Assalamu Alaikum. Greetings from icddr,b. We came from Matlab health research facilities of icddr,b. You might know that cardiovascular disease (CVD) is globally the leading cause of morbidity and mortality. The prevalence has reduced in high income countries. It is rapidly increasing in South Asian and other low and middle income countries due to a rapid epidemiologic transition resulting in high rates of risk factors. The high burden of CVD in Bangladesh is confirmed in a recent study which found that ischemic heart disease (IHD) and stroke are top two causes of years of life lost (YLLs) in Bangladesh. On the other hand, all the common behavioral, metabolic and physiological risk factors for CVD are highly prevalent in Bangladesh. To tackle this rising epidemic of CVD health facilities in low-income and middle-income countries need to be strengthened enough to provide acute management of CVD which will prevent premature deaths and disabilities. Chronic noncommunicable disease unit (CNCDU), icddr,b is going to implement a study to find out opportunities for expanding service coverage for acute vascular events at primary care level in rural Bangladesh.

**Why selected**

We are conducting a research to explore barriers to seek care during acute vascular event. This will help us to improve access to care for managing acute CVD events at primary level. As you are a policy maker/service provider/survivor / caregiver of stroke/MI, you can provide us valuable information to achieve our goal so we are inviting you to participate in this study.

**What is expected from the participants?**

If you consent to participate in this study, we will try to explore information on personal/financial/organizational barriers for accessing care during acute vascular event at primary care. We also try to explore opportunities that might improve access to care during emergency at primary care level. The interview might take your 60-90 minutes.

**Risk and benefits**

Participation in this study virtually involves no risks. You may or may not directly be benefited from participation in the study. But your contribution will enrich our knowledge of health care service delivery for acute management of CVD for rural population

**Privacy, anonymity and confidentiality**

We would like to assure you that the information collected from you including information of your health center will not be passed on to anybody else. Only the researchers of this study and Ethical Review Committee (ERC) will be able to look at the information. We do hereby affirm that privacy, anonymity and confidentiality of data/information identifying you will be strictly maintained. Each of you will have a code number and this number will be used for your identification. Your name or any information about you that could be used to identify you (find out who you are) will not be published or shared with anyone else.

**Future use of information**

The information to be collected in this study might help to gain valuable experience on developing health system interventions for preventing CVD. In case of future use of the information/data collected from this study, only anonymous or abstracted information and data may be supplied to other researchers, without any conflict with or violation of the maintenance of privacy, anonymity and confidentiality of information that identifying participants in any way.

**Right not to participate and withdraw**

Your participation is voluntary. This means that you may choose not to be in this study. You may also choose to withdraw your name from the study at any time, even after you have said that you wanted to be in the study. You don’t have to answer any question that makes you feel uncomfortable. You may ask any question about this study and we shall be happy to answer it. For further questions you may call Shyfuddin Ahmed, principal investigator of this study over telephone number 01712877165. If you feel that you have been treated unfairly or have been hurt by joining in this study you may also call M A Salam Khan, IRB Coordinator, Research Administration at icddr,b. The phone contact number of M A Salam Khan is 9827084 Ext 3206.

Do you have any questions? Yes No

Do you agree to be in the study? Yes No

Now we invite you to participate in this study. If you agree, please put your signature or your left thumbprint in the space provided below as an indication of your participation to the study.

Thank you for your cooperation.

_________________________________________________ __________________

Signature or left thumbprint of the guardian of the participant Date

_________________________________________________________ ___________________

Signature of the witness Date

________________________________________________________ ___________________

Signature of the PI or his representative (with full name & designation) Date
